# Clinical, Virologic and Immunologic Correlates of Breast Milk Acquired Cytomegalovirus (CMV) Infections in Very Low Birth Weight (VLBW) Infants in a Newborn Intensive Care Unit (NICU) Setting

**DOI:** 10.3390/v13101897

**Published:** 2021-09-22

**Authors:** Nelmary Hernandez-Alvarado, Ryan Shanley, Mark R. Schleiss, Jensina Ericksen, Jenna Wassenaar, Lulua Webo, Katherine Bodin, Katelyn Parsons, Erin A. Osterholm

**Affiliations:** 1 Department of Pediatrics, University of Minnesota Medical School, Minneapolis, MN 55454, USA; hernande@umn.edu (N.H.-A.); erick377@umn.edu (J.E.); jwassena@umn.edu (J.W.); webo0002@umn.edu (L.W.); bodin111@umn.edu (K.B.); parso366@umn.edu (K.P.); 2 Biostatistical Design and Analysis Center, University of Minnesota Medical School, Minneapolis, MN 55414, USA; shan0219@umn.edu

**Keywords:** cytomegalovirus (CMV), breast milk, post-natal CMV, CMV antivirals

## Abstract

Cytomegalovirus (CMV) infections acquired by very-low-birthweight (VLBW) infants are incompletely characterized. To examine CMV transmission in VLBW infants, we evaluated maternal DNAlactia, infant DNAemia, and presence of clinical disease in a blinded study in VLBW infants in our newborn intensive care unit (NICU). To examine these issues, 200 VLBW infants were enrolled in a surveillance study, with weekly breast milk and infant whole blood samples collected, as available. Virologic (breast milk and infant whole blood real time PCR) and immunologic (IgG, IgM, and IgG avidity) correlates were evaluated. A chart review examined whether infants had symptoms compatible with CMV disease. DNAlactia was identified in 65/150 (43%) of lactating mothers. Nine CMV infections were identified in 9/75 CMV-exposed infants (12% of exposed infants). A higher median breast milk viral load (DNAlactia) correlated with an increased likelihood of DNAemia (*p* = 0.05). Despite potential symptoms compatible with CMV infection, clinicians had not considered the diagnosis of CMV in 6/9 cases (66%). All of these infants had chronic lung disease at discharge. There was no correlation between IgG antibody titer or IgG avidity index and the likelihood of transmission or CMV disease. In conclusion, in VLBW infants receiving milk from seropositive mothers, CMV infections are commonly acquired, and are frequently unrecognized. Future studies are needed to determine whether routine surveillance for CMV of either breast milk or infant plasma is beneficial in preventing or recognizing infection.

## 1. Introduction

Human cytomegalovirus (CMV) is one of the most commonly encountered viral pathogens in newborn infants [1]. In the infant, infection occurs in the context of transplacental transmission (congenital CMV), or post-natal transmission [2]. Post-natally acquired CMV infections, which are virtually always transmitted by breast-feeding, are ubiquitous and generally of little clinical significance in term babies. In contrast, in premature infants, including very-low-birth-weight (VLBW; <1500 g) and extremely-low-birth-weight (ELBW; <1000 g) infants cared for in the Newborn Intensive Care Unit (NICU) setting, the clinical impact of breast milk-acquired CMV infection is substantial. The rates of symptomatic disease are generally low, but range considerably among studies. One review of twenty-six published studies noted up to 34.5% of premature infants that acquire such infections may have clinical manifestations, which can include hepatopathy, neutropenia, thrombocytopenia, and sepsis syndrome [3]. Another review predicted that, among 299 infants fed untreated breast milk, there would be an estimated 19% (11−32%) of VLBW babies that would acquire post-natal CMV infection, and that 4% (2−7%) of these would develop CMV sepsis-like syndrome [4].

Post-natal acquisition of CMV infection in this setting has been associated with long-term neurological consequences that can be detected via fMRI in older children when higher-level cognitive tasks are assessed [5]. Another recent study examined the prevalence of failed hearing screens, the calculated post-natal age, and somatic growth parameters at discharge in VLBW infants with post-natally acquired CMV infections (and uninfected controls) at the time of NICU discharge. In this study, newborn hearing screen (NHS) failure occurred in 45/273 (16%) infants with post-natal CMV infection, compared to 25/273 (9%) infants without post-natal CMV (*p* = 0.01), suggesting that post-natal acquisition of CMV infection in VLBW infants might contribute to the development of sensorineural hearing loss (SNHL), as is well-known to occur in the context of congenital CMV infection [6]. Other health consequences of acquisition of CMV infection in the VLBW infant include an increased risk for bronchopulmonary dysplasia (BPD), an increased requirement for vasopressor medications, a greater likelihood of intubation and mechanical ventilation, enhanced use of oxygen, and death [7]. Necrotizing enterocolitis (NEC) has also been described in association with post-natal CMV infection [8,9,10].

The variable range of symptomatic disease following perinatal CMV transmission from breast milk may relate to the absence of systematic determination of maternal CMV serostatus at delivery. Thus, without maternal CMV sero-screening, it is difficult for the neonatologist to know which infants will be at risk for acquisition of CMV in the NICU. Similarly, the lack of routine virological surveillance of infants for CMV infection in the NICU, and the relatively non-specific presentation of CMV disease in VLBW newborns, each make diagnosis challenging. Findings such as leukopenia, thrombocytopenia, transaminitis, and vital sign instability may suggest post-natal CMV infection, but might also be attributed to other common problems in premature infants, such as sepsis, lung disease, hyperalimentation, and other issues.

To address these areas of knowledge deficit, we undertook the studies described in this paper to determine the prevalence of CMV DNAlactia, the rate of transmission of CMV infection as assessed by DNAemia, and the immunological correlates associated with transmission, in a blinded study in VLBW preterm infants at the University of Minnesota NICU in Minneapolis, MN, using convenience sampling of breast milk and blood obtained in the context of routine neonatal care. A retrospective chart review was conducted to examine the clinical course of CMV infection in VLBW infants from our NICU, and to gauge whether there was evidence for acute or chronic CMV disease complications. We also evaluated whether the clinicians involved in the care of these infants had tested for and/or recognized CMV infection and disease in the course of real-time bedside care.

## 2. Materials and Methods

### 2.1. Study Population

**Figure 1:**
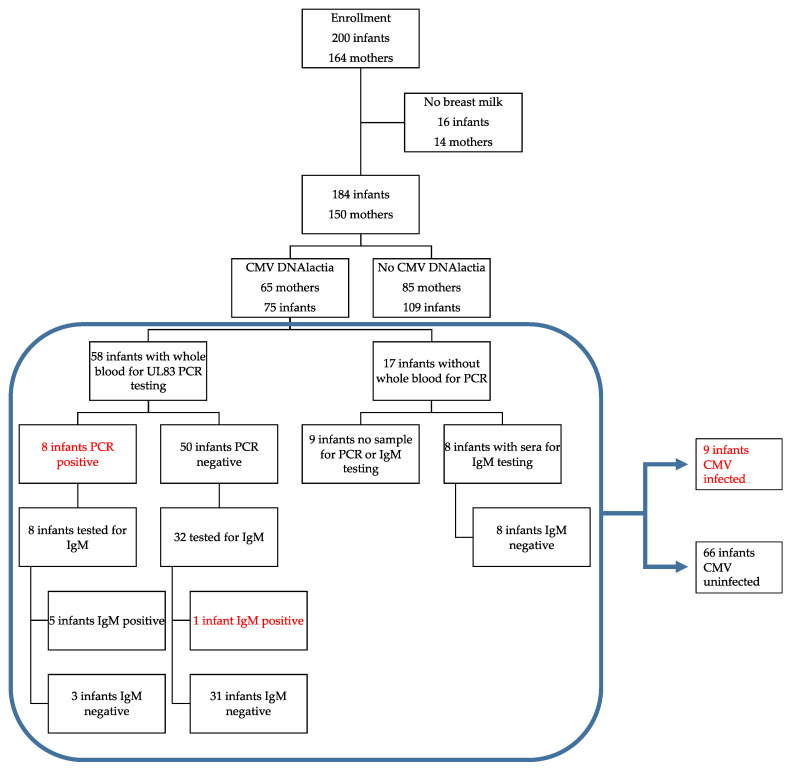
Study flow diagram. This diagram includes maternal DNAlactia, evidence for breast milk transmission of cytomegalovirus (CMV) to the infant, and the method(s) used to determinate transmission of CMV. Red font indicates infants that were determined to be CMV-infected during their NICU hospitalization.

The population that was evaluated included preterm VLBW infants (<1500 g birth weight) hospitalized in the NICU at the University of Minnesota (UMN) Masonic Children’s Hospital from October, 2014 to July, 2019, whose mothers planned to breastfeed. The UMN NICU does not as a matter of policy engage in any CMV inactivation procedures, such as freezing, although most milk is fed to babies after freeze-thaw as a matter of storage convenience. Mothers were approached during the infant’s stay for consent to participate in this study. Infants that were determined to have congenital CMV (cCMV) as a result of the standard of care were ineligible to participate. However, the study did not include testing for cCMV prior to enrollment. A total of 200 infants were consented (corresponding to 164 mothers), but for 14 mothers there were no breast milk samples available for evaluation. Hence, 150 mothers were considered evaluable for breast milk CMV shedding (DNAlactia). This corresponded to 184 infants for whom at least one breast milk sample was available, since for the 14 mothers for whom there were no breast milk samples, there were two sets of twins, and therefore a total of 16 infants for which there was no corresponding milk sample. Of the remaining 184 infants (including twin and triplet pregnancies), where at least one breast milk sample was available, this corresponded to 150 mothers. Fresh breast milk was collected on a weekly basis, depending on its availability. There was substantial variability in the number of breast milk samples collected from each mother, due to the difference in duration of infant hospitalization in the NICU and availability of the breast milk on a week-to-week basis. Leftover whole blood and serum/plasma sample remnants were obtained from the infants’ routine care, as available. In total, 996 discrete breast milk samples were obtained during the course of the study, from 150 mothers, and 1214 whole blood samples were available from 158 infants for viral load analysis by PCR. Since this was a retrospective study, none of the findings influenced clinical care. A flow chart summarizing the study is show in Figure 1.

A chart review of infants with DNAemia or positive IgM assay was conducted to identify whether the hospital course was characterized by signs or symptoms of illness that might suggest CMV disease (sepsis syndrome, elevated transaminases, leukopenia, pneumonia, vital sign instability). Discharge summaries were examined in all enrolled infants to evaluate for: (1) gender; (2) pregnancy type (singleton, twin, and triplet); (3) estimated gestational age (EGA) at delivery; (4) birth weight; (5) APGAR score; (6) head circumference at birth; (7) length of hospitalization; (8) corrected EGA at discharge; (9) survival; (10) intraventricular hemorrhage; (11) NEC; (12) oxygen therapy at 36 weeks corrected gestational age; (13) home oxygen therapy; and (14) home diuretic therapy.

### 2.2. Sample Preparation

Breast milk samples were aliquoted and stored at −20 °C until they were ready to be used. Whole blood samples were similarly stored in EDTA until processing. For PCR assays, 100 µL of unfractionated breast milk was extracted on the QIAcube (Qiagen, Hilden, Germany) using the manufacturer’s specifications for the DNeasy^®^ Blood and Tissue kit. Blood samples were purified from 100 µL using the QIAcube HT^®^ (QIAGEN), and QIAamp^®^ 96 DNA QIAcube kit according to the manufacturer’s instructions. All samples were eluted in 100 μL of PCR-grade water, and stored at −20 °C.

### 2.3. PCR Assays

Multiplex qPCR used primers described in Table 1 with slight modifications from previously published protocols [11]. The reaction was performed in a 25 μL total volume with 10 μL of template using LightCycler 480 Probes Master Mix (Roche, Basel, Switzerland) containing FastStart Taq DNA Polymerase, reaction buffer, dNTPs mix (with dUTP instead of dTTP), and MgCl2; as well as 0.4 μM primers, 0.1 μM probes, and 0.4 U/μL of uracil-DNA glycosylase (UNG). PCR was performed using the Lightcycler 480 (Roche) under these conditions: 40 °C for 10 min, 95 °C for 10 min, followed by 45 cycles of 95 °C for 10 s and 60 °C for 45 s, then a final hold step at 40 °C. A standard curve for the UL83 control was generated using 10-fold dilutions from 10^6^ to 10 copies/µL of a plasmid (UL83 fragment cloned in pCR2.1, using primers UL83_TM857F and UL83_TM1138R). The standard curve for NRAS was generated using five 10-fold dilutions starting with 200,000 to 20 pg/µL of human genomic DNA. The standard curve for NRAS PCR was generated using five, 10-fold dilutions starting with 200,000 to 20 pg/µL of human genomic DNA (Roche).

### 2.4. Serology, ELISA and Avidity Assays

Serum samples were obtained as convenience samplings from routine laboratory monitoring in the NICU undertaken as part the infants’ clinical care. The serum samples were stored at −20 °C. A total of 175 out of 200 infants had at least one serum sample (for serological assays). The total number of samples that we received was 1972. Since maternal serum samples were not available, and since infant CMV IgG serology is known to correlate well with maternal serology [12], we inferred the maternal serostatus from the infants’ serum by analyzing the earliest serum sample available to us for each infant.

Sera were tested using the Diamedix CMV IgG Enzyme Immunoassay Test Kit (ERBA Diagnostics, Miami Lakes, FL, USA) following the manufacturer’s specifications. Results obtained were expressed in semi-quantitative ELISA Units per milliliter (EU/mL). A range of 0 to <8 EU/mL was considered negative for CMV IgG; a range from ≥8 and <10 EU/mL was categorized as indeterminate; and a level of ≥10 EU/mL was considered positive.

IgG avidity was measured using the IgG CMV Diamedix assay, with slight modifications. Briefly, patient sera were diluted 1:100 in sample A diluent, according to the manufacturer’s specification. After an hour at 37 °C, the well’s contents were discarded and washed with 300 µL of Wash S buffer 3 times. Wash buffer (control buffer) was then added to one of each pair of duplicated wells; dissociating buffer (6 M urea) was added to the other duplicate well. After 5 min at room temperature, the well’s contents were discarded, and the wash procedure was repeated. The contents of the wells were discarded and 100 μL of conjugate buffer was added to the wells and incubated for 1 h at 37 °C. The contents were discarded and 100 μL of substrate solution was next added to the wells. After 20 min of incubation at 37 °C, 100 μL of stop solution was added and the OD values determined. The avidity index for each individual patient sample was calculated using the following formula: (OD for the well washed with dissociating buffer/OD for the well washed with control buffer) × 100, and was expressed as a percentage.

Qualitative detection of CMV IgM was using Gold Standard Diagnostic CMV IgM assay (GSD, 2851 Spafford Street, David, CA, USA) by following the manufacturer’s instructions. Test results were interpreted as negative, equivocal, or positive based on defined thresholds outlined in the manufacturer’s instructions for use. An index value less than 0.9 was negative, greater than or equal to 0.9 but less than 1.1 was equivocal, and greater than or equal to 1.1 was positive.

### 2.5. Statistical Analyses

**Figure 2:**
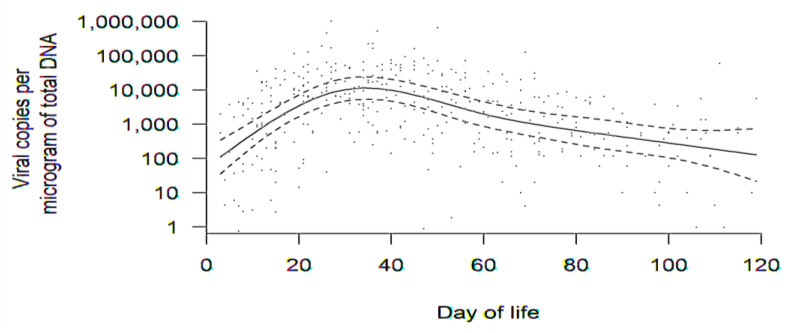
Aggregate trend in DNAlactia (viral load, copies/μg total DNA) over time (through day of life 120) for all available samples for the 65 mothers with PCR-positive breast milk. Raw data for viral load from breast milk samples were used to calculate a group average at various times, accounting for how individual averages changed over time. Data in this figure were comprised of PCR results from 380 discrete breast milk samples. The dotted lines are 95% confidence bands for the mean trend.

Statistical analyses were performed by the Biostatistical Design and Analysis Center at the University of Minnesota CTSI. We used a combination of graphs, descriptive statistics, and inferential statistics to characterize this cohort. The study was not powered to evaluate a specific hypothesis. Null hypothesis testing used Fisher’s exact test for categorical variables, and the Mann–Whitney–Wilcoxon test to compare the distributions of continuous variables. The mean trendline in Figure 2 was generated using a generalized additive model (R software version 4.0.5), with log viral load as the outcome, predicted by a 5-knot regression spline for day of life, with a random subject effect. Additional comparisons of viral load and immunological parameters were conducted using Prism^®^ 8.0 (GraphPad software).

To address the issue of the level of significance, we avoided defining “significance,” or any such bright line categorization of *p*-values, which is consistent with the recommendation from the American Statistical Association [13].

## 3. Results

### 3.1. Dynamics of DNAlactia

There were 200 infants in the sample (some of which were twins or triplets). A total of 184 infants were born to 150 mothers with evaluable breast milk samples (164 mothers consented, but breast milk was unavailable for 14 mothers). In total, 65 mothers had DNAlactia in at least one breast milk sample, corresponding to 75 infants. Thus, DNAlactia was identified in 65/150 = 43% of lactating mothers for whom comparisons could have been made. Figure 2 demonstrates the aggregate trend in DNAlactia over time for the 65 mothers. Breast milk samples were collected as convenience samples, and not as a part of a defined schedule. Therefore, in order to generate an estimate of the dynamics and patterns of DNAlactia, raw data for viral load from breast milk samples was used to calculate a group average at various times, accounting for how individual averages changed over time (to account for the variability in the number and timing of milk samples collected as convenience samples). The dotted lines demonstrate the 95% confidence band for the mean trend. Heterogeneity among individuals was noted, but the mean trend demonstrated what was estimated to be a peak viral load at approximately day 30 of lactation, with a gradual decline noted thereafter.

### 3.2. Clinical Outcomes and Comparisons in Infants of Mothers with and without DNAlactia

We next determined the impact of DNAlactia on clinical outcomes in VLBW infants in this study. A total of 75 infants from 65 mothers were exposed to CMV in the breast milk. Breast milk samples corresponding to 109 infants tested negative for CMV DNA by PCR. Table 2 is a description of the characteristics of breast milk samples by CMV results, including viral load data, for infants (not for mothers), in this study. Before analyzing the outcome between infants exposed to CMV DNAlactia (75) and infants not exposed to CMV DNAlactia (109), we wanted to compare the number of breast milk samples for each cohort. Table 2 demonstrates that the total number of evaluable breast milk samples was similar for the infants that received CMV-positive milk (75 infants), and the infants that received CMV-negative milk (109 infants), reducing the possibility of any bias in viral load comparisons based on the total number of breast milk samples analyzed in each group.

The infant discharge characteristics noted in Section 2.1, as well as the characteristics of infants receiving either CMV-positive or -negative breast milk, are indicated in Table 3. A difference in APGAR score at 1 min was noted in the DNAlactia-negative group (*p* = 0.03). It was also of interest to note racial differences with respect to the prevalence or absence of DNAlactia. Among positive samples, 67% of samples were from white mothers; on the other hand, among CMV-negative breast milk samples, an even higher percentage, 90%, was observed for white mothers (*p* < 0.01). Ethnicity-based differences were also observed, with all samples from Hispanic mothers testing positive for CMV DNA (*p* = 0.02). However, we note that this could be due to the racial and ethnic differences in CMV seropositivity among the general population as described in previous work [12]. No other differences in outcomes could be attributable to breast milk status.

### 3.3. Serological Correlates of DNAlactia

For these assays, serum samples obtained in the course of routine infant clinical care in the NICU were used. In total, 175 out of 200 infants had at least one serum sample available for assessment. The presumptive maternal IgG serostatus was inferred from the assessment of the infants’ serum. The earliest serum sample available to us for each infant was used; for 26 infants, the first sample available to us was after 31 days of age. Of the 175 for whom sera were available, 84 out of 175 (48%) were positive for IgG, and 91 out of 175 were negative for IgG (52%).

These seropositive infant samples corresponded to 72 pregnancies, with 55 singleton pregnancies and 17 twin pregnancies (89 total infants). A total of 84 of the 89 infants, corresponding to these 72 pregnancies, were seropositive for IgG; the remaining five babies, interestingly, were twin pregnancies in which the twins were discordant for CMV antibody status (i.e., one infant in the twin pair was IgG-positive, and the other infant was IgG-negative, or had no sample available for testing). For purposes of estimating maternal seroprevalence, if either twin had IgG antibody, the mother was designated as CMV-positive. Based on negative results in infant sera, the total number of mothers inferred to be CMV-seronegative was 69. For 23 mothers, no infant sera were available for testing. Thus, for pregnancies for which sera was available for testing from the corresponding infant(s), 72/141 (51%) mothers were inferred to be seropositive for CMV IgG.

Next, it was of interest to examine the correlation between the presence of antibody and the demonstration of DNAlactia in the corresponding maternal breast milk (Table 4). For the 72 evaluable CMV-seropositive mothers (with infant serum samples demonstrating IgG by ELISA), three did not have any corresponding breast milk sample available for PCR testing; 54 were positive for CMV DNA in their breast milk, and 15 were negative for CMV DNA in their breast milk. Thus, for mothers for which information was available, 54/72 inferred IgG seropositive mothers (75%) had evidence of reactivation of CMV in breast milk, whereas 15/72 (21%) did not demonstrate DNAlactia, and for 3/72 (4%), the status could not be determined because no breast milk sample was available. Three breast milk samples were positive by PCR even though the available, corresponding infant serum samples were negative for IgG antibodies (seronegative mother), possibly because of diminished IgG transfer in the context of extreme prematurity [12].

### 3.4. Infant DNAemia, CMV Disease, and Serological Correlates of Transmission

To assess for evidence of acquisition of CMV infection in infants receiving milk from women with DNAlactia, convenience samples of infant blood were examined for DNAemia by PCR. A total of 58 infants, in which breast milk status was known, had at least one blood sample available for PCR analysis. All of these infants were negative for CMV DNA on the first sample available for testing, at a median of 11 DOL and with an interquartile range (IQR) of 5–22. A total of eight of these infants exposed to CMV in breast milk developed DNAemia. Hence, the transmission rate was 14% (8/58) based on demonstrable infant DNAemia in infants for whom we had both infant virological data from blood samples (PCR) and breast milk PCR data. Blood samples from the remaining 50 infants receiving CMV PCR-positive breast milk were uniformly negative by infant whole blood PCR, although one PCR-negative infant in this group had a positive IgM serology (described below). There were eight infants for whom we were able to perform IgM testing, but for whom we did not have any whole blood samples for PCR. Thus, when PCR and/or IgM testing were evaluated, CMV infection was identified in 9/66 evaluable infants (13.6%).

**Figure 3:**
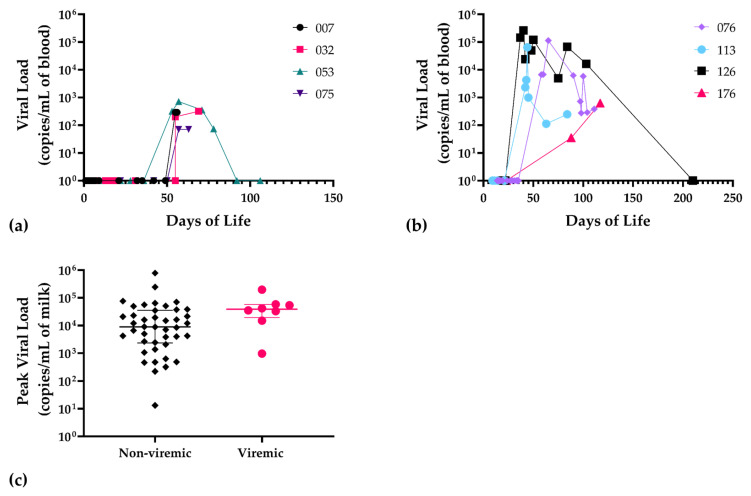
Patterns of DNAemia in infants and corresponding maternal breast milk viral load in VLBW infants acquiring infection. (**a**) Temporal sequence of DNAemia showing viral blood load in infants 007, 032, 053, 075, and (**b**) infants 076, 113, 126, and 176; (**c**) viral load in maternal breast milk samples corresponding to the remaining 50 exposed, non-viremic infants (black diamond symbols) compared to viremic infants (*n* = 8; red circles), *p* = 0.05, Mann–Whitney. For statistical comparisons, negative samples were assigned a value of 1 copy/mL of blood.

As a control, whole blood PCR was performed on a subset of available samples from all infants that had received PCR-negative breast milk. These results were uniformly negative (data not shown). Notably, there was no infant with a positive blood PCR that did not receive PCR-positive breast milk. The temporal sequence of positive PCR signals in infant blood samples was determined (Figure 3a,b) and, notably, all infants had negative samples prior to developing DNAemia, compatible with post-natal CMV acquisition. DNAemia was correlated with the mean magnitude of maternal DNAlactia in maternal breast milk samples (Figure 3c). For non-viremic infants, the median corresponding breast milk viral load was 8985 copies/mL of blood (c/mL bool), IQR (2350–35,650), mean 41,390 c/mL blood, SD 124,182; for viremic infants, median corresponding breast milk viral load was 38,700 c/mL blood, IQR (19,500–57,175), mean = 54,459 c/mL blood, and SD 60,627. The *p* value for this was *p* = 0.05. Interestingly, two of the viremic infants each had a twin with a negative CMV blood PCR (i.e., two twin pairs with discordant CMV results). In each case, the twin was consistently PCR-negative in all samples. Thus, in each of these discordant twin pairs, there was evidence of breast milk transmission in one baby with attendant DNAemia, but not both of the twins.

**Figure 4:**
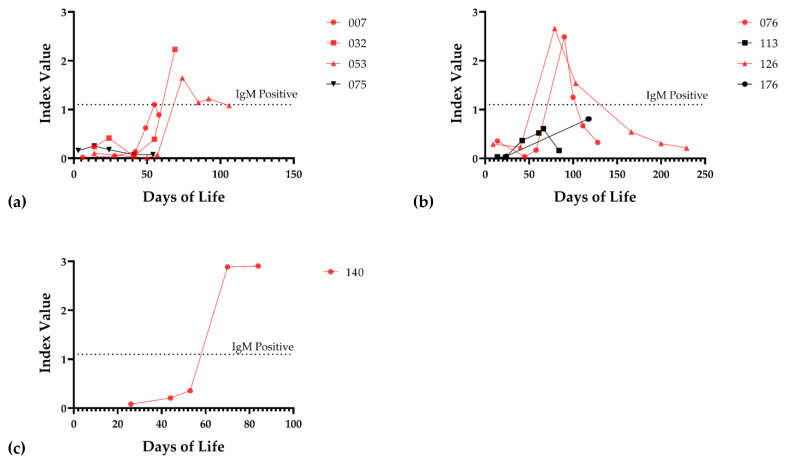
IgM response of infected infants. (**a**) IgM response of viremic infants 007, 032, 053, and 075. (**b**) IgM response of viremic infants 076, 113, 126, and 176. (**c**) IgM response of infant 140. Red lines indicate that the infant mounted a positive IgM response at some point during the hospital stay; the threshold of detection for the IgM assay is shown (dotted line).

To further examine for possible CMV infection in the neonate that could not be identified by whole blood PCR (due to limited availability of samples or timing of sample acquisition), a subset of serum samples was assayed for IgM antibodies. We tested IgM from 40 infants exposed to CMV-positive breast milk at a median DOL of 57, IQR (45.5–88.5). For controls, we used 36 infants that were not exposed to CMV-positive breast milk. We also examined available sera from infants with documented DNAemia for the appearance of IgM antibodies (Figure 4a,b). One non-viremic infant exposed to CMV in the breast milk was positive for IgM antibodies (Figure 4c). The serum sample from the infant with positive IgM was at 84 DOL. To further examine the possibility that this baby was infected with CMV in the hospital course, we also tested serum samples for IgM at 26, 44, 53, and 70 days of life, in addition to the day 84 sample (Figure 4c). The infant was negative for IgM at 26, 44, and 53 days of life, but positive at 70 and 84 days of life, which suggested acquisition of an infection at approximate DOL 50. However, the only infant whole blood sample available to us to evaluate for DNAemia for this particular infant had been obtained at DOL 25, and this sample was negative. In summary, we concluded that 8/58 (13.8%) infants were infected with CMV, with breast milk as the likely source, during their NICU course, using PCR as the defining variable for transmission, from 58 evaluable infants. When IgM serology was included, 9/66 (13.6%) of tested infants had evidence of acquisition of CMV from breast milk in the NICU. As a control, for the 17 infants receiving CMV-positive breast milk for whom no neonatal blood sample was available for DNAemia testing, eight available infant serum samples were tested for IgM antibodies; all were negative (Figure 1).

To further evaluate outcomes of CMV-infected infants, we evaluated the characteristics of infants (*n* = 75, delivered to 65 mothers) exposed to CMV-positive milk. We compared infants who were demonstrated to have CMV infection (*n* = 9) by PCR or IgM assay to the infants known to be exposed to CMV PCR-positive breast milk who did not demonstrate evidence of infection, either by testing performed during their hospitalization (*n* = 66) or by PCR testing of infant blood samples. Table 5 reviews these comparisons. No differences in gender, gestational age, birth weight, APGAR score, or head circumference at birth were noted.

To further characterize disease outcomes, we examined whether the clinicians involved in the care of these VLBW infants at the bedside had identified signs or symptoms compatible with CMV infection or disease during the NICU hospitalization, by examining the hospital charts of these nine infants. Possible associated symptoms were identified in most infants, although it was not easy to discern whether symptoms were specifically attributable to CMV infection, as opposed to other common diseases of prematurity. For these infants (Table 6), it was also noted that CMV had been considered and confirmed in three of these nine infants; CMV surveillance PCR testing of blood in infant 140 was negative, but the diagnosis was not strongly considered (although IgM testing done in the context of this study was positive, as described in the text). Congenital CMV was excluded by testing in four infants; three in the immediate newborn period, and one by retrospective testing of the archived newborn dried blood spot [14]. One infant was treated with a six-month course of ganciclovir/valganciclovir. Of note, three babies in this group failed their newborn hearing screens (NHS) prior to discharge.

We next evaluated the outcomes at discharge in the nine infants with evidence of breast-milk-acquired CMV infection (Table 7). Outcomes examined included duration of hospitalization prior to discharge; presence of intraventricular hemorrhage (IVH); medical and surgical necrotizing enterocolitis (NEC); oxygen requirement at 36 weeks EGA; home diuretic therapy; and home oxygen therapy. There were no large differences between the infected and uninfected infants for IVH and NEC (data not shown). However, the length of hospitalization for the CMV-infected infants was higher (*p* = 0.0177). The percentage of infants that needed oxygen at 36 weeks GA was also statistically different (*p* = 0.0118). Moreover, even at discharge, the CMV-infected infants still required supplemental oxygen at a higher rate (77.8%, 7/9) than their non-infected counterparts (24.24%, 16/66; *p* = 0.0028).

**Figure 5:**
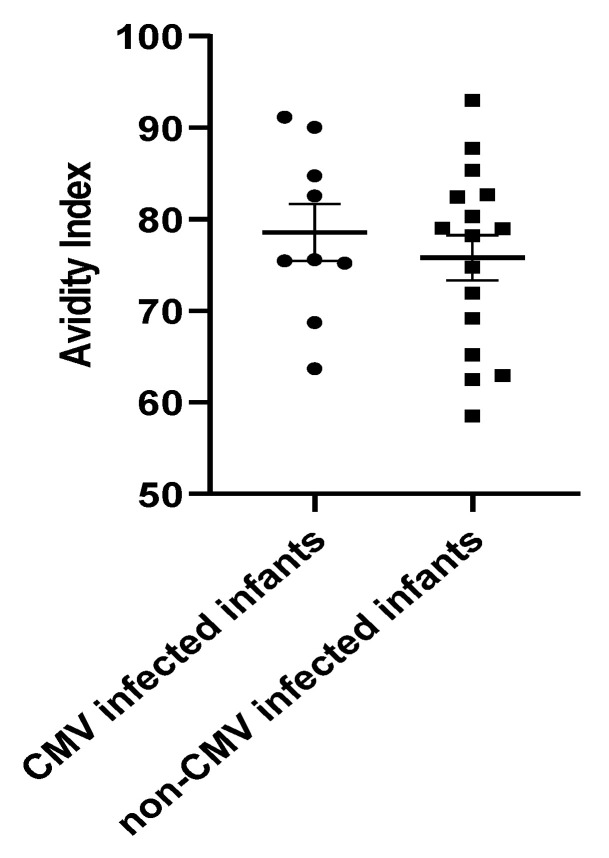
IgG Avidity Index for CMV-infected (*n* = 9) and control infants (*n* = 16). The bold line represents the mean and error bars of the SEM. The median for CMV-infected infants was 75.60% with an IQR of 71.98–97.16. The median for the non-CMV-infected infants was 78.59% with an IQR of 66.19–93. No evidence for the impact of IgG avidity on the risk of infection was observed. Mann–Whitney test, *p* = 0.6.

Next, it was of interest to examine the correlation between the ELISA IgG titer and IgG avidity index of the infants who had evidence of acquisition of CMV infection in the NICU. For the mothers of these nine infants (eight infants with DNAemia, and one infant with acquisition of IgM antibodies) antibody titers were calculated, using the earliest available serum samples obtained from the infants. The infant ELISA titer (inferred maternal titer) in those with documented CMV infections were compared to those inferred for mothers (*n* = 63 mothers) in which no evidence of transmission of infection was identified in their infants (by either PCR testing of infant whole blood, or IgM testing). No differences in IgG ELISA titer were noted comparing these two groups (data not shown). We next evaluated whether differences in IgG avidity index were present. The goal was to examine whether the infected infants had lower IgG avidity than controls without evidence of CMV infection (negative infant whole blood PCR and negative IgM serology). We selected the earliest available serum samples for the nine infected infants and the 16 CMV breast-milk-exposed but uninfected controls, to attempt to best represent the inferred maternal IgG avidity index. For controls, we used serum from infants that had similar IgG levels to the infected infants. The median DOL for the serum samples was 14, with an IQR [11–21.5]. Controls were selected to match, as possible, with the following parameters: mother peak breast milk viral load; gestational age; and birth weight. We did not see any difference in IgG avidity between the infected infants and controls (Figure 5).

## 4. Discussion

In this paper, we report on a study of a total of 200 VLBW infants born to 164 mothers who were hospitalized in the NICU at the UMN. The overall prevalence of DNAlactia from mothers with breast milk samples was 65/150 = 43.3%. We examined the temporal sequence of DNAlactia in breast milk samples. A limitation of this study was that we were not able to collect timed, serial breast milk samples; rather, this was a convenience set of samples collected throughout the course of NICU hospitalization. In order to characterize trends in DNAlactia, we examined aggregate trends (Figure 2), focusing on average individual changes over time. The mean trend demonstrated a peak viral load approximately at DOL 30, with a gradual decline thereafter, with a unimodal curve distribution. Other studies have found similar viral shedding kinetics, where viral shedding begins during the first week of lactation, with reports variably noting a peak viral load at 4–8 weeks postpartum [15,16,17,18]. We observed a gradual decline of viral load through DOL 120. Different studies report variability in the duration of DNAlactia during normal lactation in CMV-seropositive mothers. In a review of published reports, it was noted that several studies report a significant reduction in DNAlactia after 9–12 weeks of lactation with a cessation of viral shedding at 3 months of lactation; on the other hand, CMV shedding is detectable as far out as nine months after delivery in other studies [19].

Overall, it has been estimated that up to 96% of CMV-seropositive women will reactivate CMV and shed viral DNA in breast milk in the post-partum period [3,20]. Previous studies demonstrated a high correlation coefficient for comparisons of maternal and neonatal CMV IgG [12]. Unfortunately, we did not have serum samples available from mothers, so we were unable to perform these correlative analyses. We did note that infant sera were CMV IgG-positive in samples corresponding to 72 mothers (there were three instances for the 75 CMV-positives for which the serological status could not be determined, since no infant sera were available for testing), and negative for 69 mothers (total available for serological testing), for an inferred seroprevalence of 72/141 (51%). We also observed CMV PCR-positive breast milk in samples corresponding to three lactating mothers with negative IgG ELISA results in corresponding infant sera, perhaps related to the sub-optimal transfer of maternal IgG in the setting of extreme prematurity [12]. Future studies will require maternal serological samples to further explore associations between potential antibody correlations associated with the presence or absence of DNAlactia.

We examined whether there was a relationship between an infant receiving CMV-positive breast milk and the clinical outcome, irrespective of the infant’s infection status (Table 3). We found no strong evidence of a relationship between an infant being fed CMV-positive breast milk and complications of prematurity, including NEC, IVH, chronic lung disease, and microcephaly. We next reanalyzed the clinical impact based on whether infants had evidence of acquisition of CMV infection during their NICU hospitalizations. We focused on infants (*n* = 75, delivered to 65 mothers) that were exposed to CMV-positive milk and examined for DNAemia in whole blood samples (Figure 3), and IgM antibody to CMV in serum (Figure 4), collected during the course of hospitalization. Baseline clinical characteristics (gender, gestational age, birth weight, APGAR score, head circumference at birth) of infected (*n* = 9) and control infants (Table 4) did not demonstrate any demographic differences.

We next examined whether a correlation between breast milk viral load and infection could be identified in viremic infants. Reduction in maternal breast milk CMV viral load has been proposed as a strategy to reduce the risk of transmission in low-birth-weight infants [21], although the role of viral load as a risk factor for an increased likelihood of transmission remains incompletely defined [4]. One study of CMV viral loads in milk whey demonstrated higher levels in CMV-transmitting mothers than in non-transmitting mothers, and attempted to define a threshold level of DNAlactia that correlated with transmission of CMV [22]. However, in a prospective, multi-center birth cohort study of >500 VLBW infants, a viral load cutoff below which breast-milk-acquired CMV infections did not occur could not be identified [23]. When we compared the viral load in CMV-exposed infants with DNAemia to controls, we noted a statistically suggestive trend (Figure 3c) toward a higher magnitude of DNAlactia in DNAemic infants (*p* = 0.05), but conclusions are limited because of the small number and convenience nature of the samples we tested. Future studies focused on time-matched, prospective comparisons of breast milk viral load and acquisition of infection are warranted, and should include evaluation of the total number of days of breast-feeding, as a measure of cumulative CMV exposure. Exclusivity of breast feeding may be a parameter worth examining as an additional risk factor for transmission, since mixed modes of feeding are known to increase the risk of post-natal HIV transmission by breast milk [24].

We assessed whether the infants that we identified with breast-milk-acquired infections had any evidence of adverse clinical outcomes, compared to exposed but uninfected controls. It was of interest that in four of the infants, congenital infection had been ruled out. Although the other infants in this group had not been formally tested for congenital CMV, we think it is unlikely that these CMV infections had been acquired before delivery, given that there is no definitive evidence that the prevalence of infection is higher in VLBW preterm infants than in term babies [25,26]. Interestingly, all infants had symptoms that could have included CMV infection in the differential diagnosis (sepsis evaluations, oxygen requirements, thrombocytopenia; Table 6), although other explanations were difficult to exclude as is always the case in neonatology practice. We examined outcomes including IVH, NEC, length of hospitalization, use of oxygen at 36 weeks corrected GA, need for home oxygen, and need for home diuretics. Consistent with past observations in other reports [6,21], the length of hospitalization for the CMV-infected infants was higher (*p* < 0.05; Table 6). We also observed an increased need for O_2_ therapy both at 36 weeks of corrected GA (*p* < 0.05) and at the time of home discharge (*p* < 0.01). Previous reports have noted an association between post-natal breast-milk-acquired CMV and lung disease, including BPD [6,7,27,28,29]. The role of perinatal breast-milk-acquired CMV in the pathogenesis of long-term disabilities, including SNHL, remains incompletely defined. Although a number of studies have not suggested any long-term neurologic or hearing disabilities associated with post-natally-acquired CMV infections [15,26], an analysis of hospital discharge information from the Pediatrix Medical Group identified CMV infection as a risk for referral on the newborn hearing screen, suggesting a potential role for infection in the development of SNHL [6], and other long-term neurological injuries associated with post-natal CMV acquisition have been observed [5].

Finally, we asked whether there were any discernable host immune features that correlated with the acquisition of CMV infection in the nine infants we identified with breast-milk-associated transmission. It is of interest that not all VLBW infants receiving CMV-positive breast milk become infected; indeed, published estimates suggest that no more than 20–40% of exposed infants will acquire infection in the NICU setting [2]. Studies have attempted to identify immunologic factors that predict breast milk transmission, including T cell, cytokine, and humoral (IgG) responses, but no clear correlates have emerged [2,30]. Although the CMV IgG avidity index has been inversely correlated to milk CMV load in breast milk, there have been no correlations between avidity index and the likelihood of breast-milk-mediated transmission [20,31]. Since we did not have maternal sera available for serological testing, we used the first available post-natal serum samples of infants in our study to infer both the total ELISA antibody titer and the maternal CMV IgG index in CMV seropositives. Based both on total IgG titer as well as avidity index, we did not identify any association between the magnitude of the serologic response and the magnitude of DNAlactia in corresponding CMV PCR-positive breast milk samples. In addition, we identified CMV IgG antibody-positive sera in infants corresponding to PCR-negative breast milk. Since these women were, by definition, CMV-seropositive, we examined whether there was any correlation between IgG titer and/or avidity index and the absence of DNAlactia. Continued study is required to identify potential correlates of immunity, such as T cells [32] or cytokines [33], that might be associated with a reduced risk of lactation-associated post-natal CMV transmission. Efforts are also required to investigate whether CMV strain variation correlates with breast-milk-mediated transmission risk [34,35]. Notably, three infants in our series of nine patients with post-natal CMV infection failed their hearing screens before NICU discharge. Further studies will be warranted to gauge the impact of post-natal breast-milk-acquired CMV on SNHL.

Our study had several limitations. The analysis was based on convenience samples, and not a longitudinally collected cohort of samples. Ideally, timed serial samples would be maintained for a longitudinal study, but for this study, we were limited to leftover plasma, whole blood, serum, and breast milk used in the context of routine care and routine laboratory monitoring. We were also limited by the lack of maternal serological samples. Finally, our study was limited in its ability to identify CMV infection because of our reliance on whole blood PCR and, in one instance, IgM testing to identify the acquisition of CMV infection. Saliva and/or urine sampling is considered to be the “gold standard” for CMV diagnosis in the neonate [36], and it is likely we may have missed CMV infections in VLBW infants receiving CMV-positive breast milk. Despite these limitations, we were able to identify nine CMV infections in 75 infants receiving CMV-positive breast milk, with an overall transmission rate of 12%.

The questions engendered by breast-milk-acquired CMV infections in the NICU setting remain unresolved. Extensive evidence documents the short-term morbidities that can be associated with such infections [2,3,18,19,37]. Although the information is incomplete, there is increasing evidence that such infections in VLBW increase the risk of long-term chronic lung disease, and possibility SNHL, as well as other neurodevelopmental disabilities [5,6,7,29]. Considerations for mitigating risk have been the subject of a recent review [2], and have included freezing or pasteurization of milk; maternal serological screening to identify CMV-seropositive women at risk for reactivation of CMV during lactations; actual testing of breast milk itself before feeding, using rapid, point-of-care testing platforms; or the addition of antiviral factor, including an antibody, to inactivate a virus without destroying the salutary components of breast milk (which occurs with pasteurization and, to a lesser but important extent, freezing). We took no special precautions to prevent CMV transmission in our NICU. Breast milk is often frozen as a matter of convenience, but there are limited and contradictory data about the efficacy of that intervention in preventing transmission [2]. To a certain amount, freeze-thawing will potentially reduce the transmission rate [4], but we were not in a position to analyze that question in the current study. In future studies, we hope to incorporate a measurement of maternal serological response to CMV to better understand the kinetics and impact of an antibody response on neutralization of a virus in blood and breast milk [38]. As vaccines approach licensure for CMV [39], it will be of particular interest to examine the impact of a maternal vaccine-induced antibody on CMV disease in this high-risk patient population.

## 5. Conclusions

In our study of VLBW infants in a NICU population in Minneapolis, Minnesota, we made the following observations:

1. The rate of virolactia due to CMV reactivation in nursing mothers, without regard to serostatus, in our total population was 65/150 or 43%.
2. The estimated (inferred) seroprevalence for CMV antibodies in lactating mothers, for pregnancies in which neonatal sera were available for testing, was 72/141 (51%) seropositive for CMV IgG.
3. Among women inferred to be seropositive based on analysis of newborn sera, the rate of viral reactivation (virolactia) in breast milk was 75% (54/72).
4. Among CMV breast-milk-exposed infants for whom infant blood was available for analysis (PCR and/or IgM serology), the overall transmission rate was 9/66 (13.6%).
5. There was no correlation between the IgG avidity index in infants with documented post-natal transmission and controls.
6. All infants with post-natal CMV infection had signs, symptoms, and/or laboratory abnormalities potentially consistent with CMV infection.
7. In nine infants with CMV infection documented by PCR of blood samples (or in one instance, positive IgM antibodies), the diagnosis of CMV was confirmed in “real-time” by clinicians in only three instances; one infant was treated with nucleoside antivirals.
8. Infants with post-natal CMV infection had longer hospitalizations; demonstrated increased oxygen requirement at 36 weeks EGA and at discharge; and had an increased need for diuretic therapy, compared to controls.
9. More knowledge is needed about the short- and long-term risks associated with breast-milk-acquired CMV infections in VLBW infants, as well as about interventional strategies to identify mother–infant dyads at risk and to prevent transmission to the newborn.

## Figures and Tables

**Table 1:** Primer and probe sequences used for PCR detection of CMV genome (UL83 gene).

Primer Designation	Sequence
UL83_TM857F	GGACACAACACCGTAAAGC
UL83_TM1138R	GTCAGCGTTCGTGTTTCCCA
UL83_TM917Pr	CFR610-CCCGCAACCCGCAACCCTTCAT-BHQ2
nras_TM360F	GCCAACAAGGACAGTTGATACAAA
nras_TM438R	GGCTGAGGTTTCAATGAATGGAA
nras_TM384Pr	FAM-ACAAGCCCACGAACTGGCCAAGA-BHQ1

**Table 2:** Characteristics of breast milk samples by CMV PCR results. Data shown are for 184 infants born to 150 mothers (for whom breast milk was available for PCR testing). In total, 75 infants were exposed to CMV-PCR-positive milk (41%) and 109 received PCR-negative milk (59%).

Characteristics of Breast Milk Samples by CMV Results		
	Positive (*n* = 75)	Negative (*n* = 109)
Number of Breast Milk Samples		
Median	6.0	5.0
Q1; Q3	3.0; 9.0	3.0; 9.0
Peak Viral Load, Copies/mL		
Median	12,300.0	NA
Q1; Q3	2440.0; 38,600.0	NA
Peak Viral Load, Copies/μg DNA		
Median	17,395.8	NA
Q1; Q3	5440.7; 107,182.3	NA

**Table 3:** Characteristics of infants receiving breast milk from mothers with DNAlactia (positive) and without CMV DNA in breast milk (negative), using infant as unit of analysis. Fisher’s exact test was used for categorical variables; Mann–Whitney–Wilcoxon used for continuous variables.

Patient Characteristics/Outcomes by Breast Milk CMV Results			
	Positive (*n* = 75)	Negative (*n* = 109)	*p*-Value
**Gender**			0.45
Female	38 (51%)	48 (44%)	
Male	37 (49%)	61 (56%)	
**Race**			<0.01
American Indian or Alaska Native	0 (0%)	1 (1%)	
Asian	11 (15%)	2 (2%)	
Black or African American	2 (3%)	4 (4%)	
Patient Refusal	5 (7%)	1 (1%)	
Unknown	7 (9%)	3 (3%)	
White	50 (67%)	98 (90%)	
**Ethnicity**			0.02
Hispanic or Latino	4 (5%)	0 (0%)	
Non-Hispanic	69 (92%)	109 (100%)	
Unknown	2 (3%)	0 (0%)	
Pregnancy type *			0.22
Singleton	55 (85%)	63 (74%)	
Twin	10 (15%)	20 (24%)	
Triplets	0 (0%)	2 (3%)	
**Gestational age at birth**			0.55
Median	28.1	28.4	
Q1, Q3	26.0, 29.8	26.7, 29.9	
**Birth Weight**			0.36
Median	1020.0	1060.0	
Q1, Q3	760.0, 1210.0	780.0, 1280.0	
**APGAR at 1 min**			0.03
Median	5.0	6.0	
Q1, Q3	3.0, 7.0	3.0, 8.0	
**APGAR at 5 min**			0.75
Median	8.0	8.0	
Q1, Q3	7.0, 8.8	7.0, 9.0	
**OFC at birth**			0.34
Median	24.5	25.0	
Q1, Q3	22.6, 26.5	23.0, 27.0	
**Length of hospitalization (days)**			0.21
Median	72.0	70.0	
Q1, Q3	50.5, 109.0	47.0, 93.2	
**Corrected gestational age at discharge**			0.15
Median	39.0	38.3	
Q1, Q3	36.9, 41.9	36.9, 40.4	
**Status at discharge**			0.84
Deceased	3 (4%)	7 (6%)	
Discharged home	66 (88%)	93 (86%)	
Transferred to another facility	6 (8%)	8 (7%)	
**Intraventricular hemorrhage**			0.77
No	69 (92%)	100 (93%)	
Yes	6 (8%)	7 (7%)	
**NEC (medical management)**			0.74
No	70 (93%)	103 (95%)	
Yes	5 (7%)	5 (5%)	
**NEC (surgical management)**			0.65
No	74 (99%)	105 (97%)	
Yes	1 (1%)	3 (3%)	
**Oxygen requirement at 36 weeks**			0.65
No	40 (53%)	53 (50%)	
Yes	35 (47%)	54 (50%)	
**Home oxygen requirement**			0.12
No	52 (69%)	62 (57%)	
Yes	23 (31%)	46 (43%)	
**Home diuretic requirement**			0.15
No	63 (84%)	80 (74%)	
Yes	12 (16%)	28 (26%)	

* For pregnancy type, numbers are provided for unique mothers, not infants.

**Table 4:** Association of serostatus (infant sera used to infer maternal seroprevalence) and DNAlactia (maternal breast milk samples). Serostatus was inferred by assay of infant IgG using the first sample available on the newborn infant, and this was compared to the presence of CMV DNAlactia measured by PCR in the corresponding breast milk sample.

	CMV DNA Breast Milk			
CMV IgG Status	Positive	Negative	No Sample	Total
Positive	54	15	3	72
Negative	3	63	3	69
No Sample	8	7	8	23
**Total**	65	85	14	164

**Table 5:** Baseline characteristics of infants exposed to CMV through breast milk. Data are median [interquartile range] or N (%). Fisher’s exact test used for categorical variables; Mann–Whitney test used for continuous variables.

	CMV–Non-Infected ^1^	CMV-Infected ^2^	*p*-Value ^3^
N per group	66 (88%)	9 (12%)	NA
Gender (male %)	31 (47%)	6 (67%)	0.31
Birth Gestational Age (weeks)	28 (25.75−30)	26 (25.5−28)	0.30
Birth Weight (g)	1040 (707.5−1250)	850 (745−1030)	0.35
Apgar at 1 min	5 (3−7)	6 (2−7)	0.97
Apgar at 5 min	8 (7−8.5)	8 (6−9)	0.95
Birth OFC ^4^ (cm)	25.40 (22.58−26.53)	24 (22.15−24.25)	0.28
Ethnicity			0.99
Hispanic	4 (6%)	0 (0%)	
Non-Hispanic	60 (91%)	9 (100%)	
Unknown	2 (3%)	0 (0%)	
Race			0.47
Asian	11 (17%)	0 (0%)	
Black or African American	2 (3%)	0 (0%)	
Patient Refusal	5 (8%)	0 (0%)	
Unknown	5 (8%)	2 (22%)	
White	43 (65%)	7 (78%)	

^1^ Based on the chart review. ^2^ Positive infant whole blood PCR or IgM. ^3^ Fisher’s exact test used for categorical variables; Mann–Whitney test used for continuous variables. ^4^ OFC, occipital frontal circumference.

**Table 6:** Characteristics of infants with post-natal infection (*n* = 9) and signs and symptoms compatible with infection. Infants 053 and 176 each had a twin that tested negative for CMV by whole blood PCR and IgM.

Study ID’	Gestational Age	Birth Weight	Congenital CMV-Tested	Clinical Evaluation for CMV Infection	First Detection of DNAemia Gestational Age (Day of Life, DOL)	Measured Peak DNAemia (copies/mL)	Possible Associated Symptoms
007	29 w, 3 d	794 g	Yes—negative	No	37 w, 2 d (55)	292	Thrombocytopenia; chronic lung disease
032	27 w, 5 d	1130 g	Yes—negative	No	35 w, 4 d (55)	318	Thrombocytopenia; chronic lung disease
053 ^1^	26 w	930 g	No	No	33 w, 4 d (53)	715	Sepsis syndrome; chronic lung disease
075	27 w, 4 d	900 g	No	No	35 w, 5 d (57)	71	Sepsis syndrome; chronic lung and renal disease
076 ^2^	24 w, 4 d	740 g	Yes—negative	Yes—positive	32 w, 6 d (58)	114,000	Thrombocytopenia, apnea, DNAemia ^5^
113	26 w	750 g	No	Yes—positive	32 w (42)	64,700	Thrombocytopenia ^5^
126	30 w, 2 d	1435 g	Yes—negative ^3^	Yes—positive	35 w, 4 d (37)	265,556	Sepsis syndrome; chronic lung disease ^5^
176 ^1^	26 w, 6 d	850 g	No	No	39 w, 3 d (88)	620	Sepsis syndrome; tracheitis; chronic lung disease
140 ^4^	25 w, 4 d	660 g	No	Yes ^6^	Positive IgM, DOL 70	NA	Sepsis syndrome; chronic lung disease

^1^ PCR-negative twin. ^2^ Treated with ganciclovir/valganciclovir. ^3^ Negative newborn dried blood spot for CMV. ^4^ CMV infection identified by IgM serology only. ^5^ Referred on NHS. ^6^ This baby was evaluated at DOL 25 and 40 by CMV PCR of whole blood, but was negative.

**Table 7:** Outcomes of infants with evidence of breast-milk-acquired CMV infections at discharge compared to controls. CMV infections were identified by retrospective analysis of infant convenience samples of whole blood (PCR) and serum (IgM assay) collected during course of hospitalization (see text for details).

	CMV-Uninfected	CMV-Infected	*p*-Value ^1^
N	66 (88%)	9 (12%)	
Length of hospitalization (days)	71 (48–103)	112 (72.50–179)	0.0177
O_2_ requirement at 36 weeks			
Yes	28 (42.4%)	8 (88.9%)	
No	38 (57.6%)	1 (11.1%)	0.0118
Home O_2_ therapy			
Yes	16 (24.2%)	7 (77.8%)	
No	50 (75.8%)	2 (22.2%)	0.0028
Home diuretic therapy			
Yes	9 (13.6%)	4 (44.4%)	
No	49 (74.2%)	5 (55.6%)	0.0431

^1^ *p*-value from Fisher’s exact test.

## Data Availability

Data from this study are stored in a secured site maintained by the UMN Center of Excellence for HIPAA Data (https://it.umn.edu/services-technologies/box-secure-storage; accessed on 14 September 2021). Data are available on request.
